# Unmasking a Rare Pelvic Masquerader: Aggressive Vulvovaginal Angiomyxoma With Perineal Herniation

**DOI:** 10.7759/cureus.98244

**Published:** 2025-12-01

**Authors:** Niranjan Kumar, Pradosh Kumar Sarangi, Kondaveeti Nikhileswar, Mona Lisa, Amiy Arnav, Satya Ranjan Patra

**Affiliations:** 1 General Surgery, All India Institute of Medical Sciences, Deoghar, Jharkhand, IND; 2 Radiodiagnosis, All India Institute of Medical Sciences, Deoghar, Jharkhand, IND; 3 Pathology and Laboratory Medicine, All India Institute of Medical Sciences, Deoghar, Jharkhand, IND; 4 Surgical Oncology, All India Institute of Medical Sciences, Deoghar, Jharkhand, IND

**Keywords:** aggressive angiomyxoma, histology, mri, pelvic mass, perineal herniation, swirling pattern, vulvovaginal mass

## Abstract

Aggressive angiomyxoma (AAM) is a rare, locally invasive, slow-growing low-grade mesenchymal tumor most frequently found in reproductive-age females with a high propensity for recurrence. AAM poses diagnostic challenges due to its indolent course, non-specific imaging appearance, and frequent misdiagnosis as more common entities like Bartholin cyst, lipoma, hernia, leiomyoma, etc. Accurate diagnosis before surgery is crucial to prevent recurrence. We present a case of AAM in a 47-year-old female with perineal herniation showing typical imaging features, including T2 swirling or laminated pattern, and correlated with histopathology post-surgical resection. The patient remained recurrence-free 12 months post-resection. This case reinforces the pivotal role of imaging, especially MRI, in preoperative diagnosis and surgical planning to minimize the likelihood of incomplete resection and recurrence.

## Introduction

Aggressive angiomyxoma is a rare, benign, slow-growing, locally infiltrative, low-grade mesenchymal tumor commonly seen in reproductive-age women. Steeper and Rosai first described it in 1983 [1]. Although described typically in reproductive-age females, a few cases in the scrotum or spermatic cord in males were reported [2].

Aggressive angiomyxoma often has a large pelvic component located deep in relation to the pelvic diaphragm, which makes it difficult to detect during physical examination and is often mistaken for Bartholin’s cysts, vaginal cysts, lipomas, leiomyoma, or perineal hernias [3]. In all cases, diagnostic certainty can be achieved only by histology [4].

Surgical resection is the mainstay of treatment. The infiltrative nature of the tumor makes it difficult for surgeons to achieve a negative surgical margin, leading to frequent recurrences in up to 35% of patients [5]. Estrogen and progesterone receptor-positive tumors can show treatment response to GnRH agonists both preoperatively and after recurrence [5,6].

We present a case of aggressive vulvovaginal angiomyxoma with perineal herniation with classic imaging features and correlating with histopathological examination.

## Case presentation

**Figure 1:**
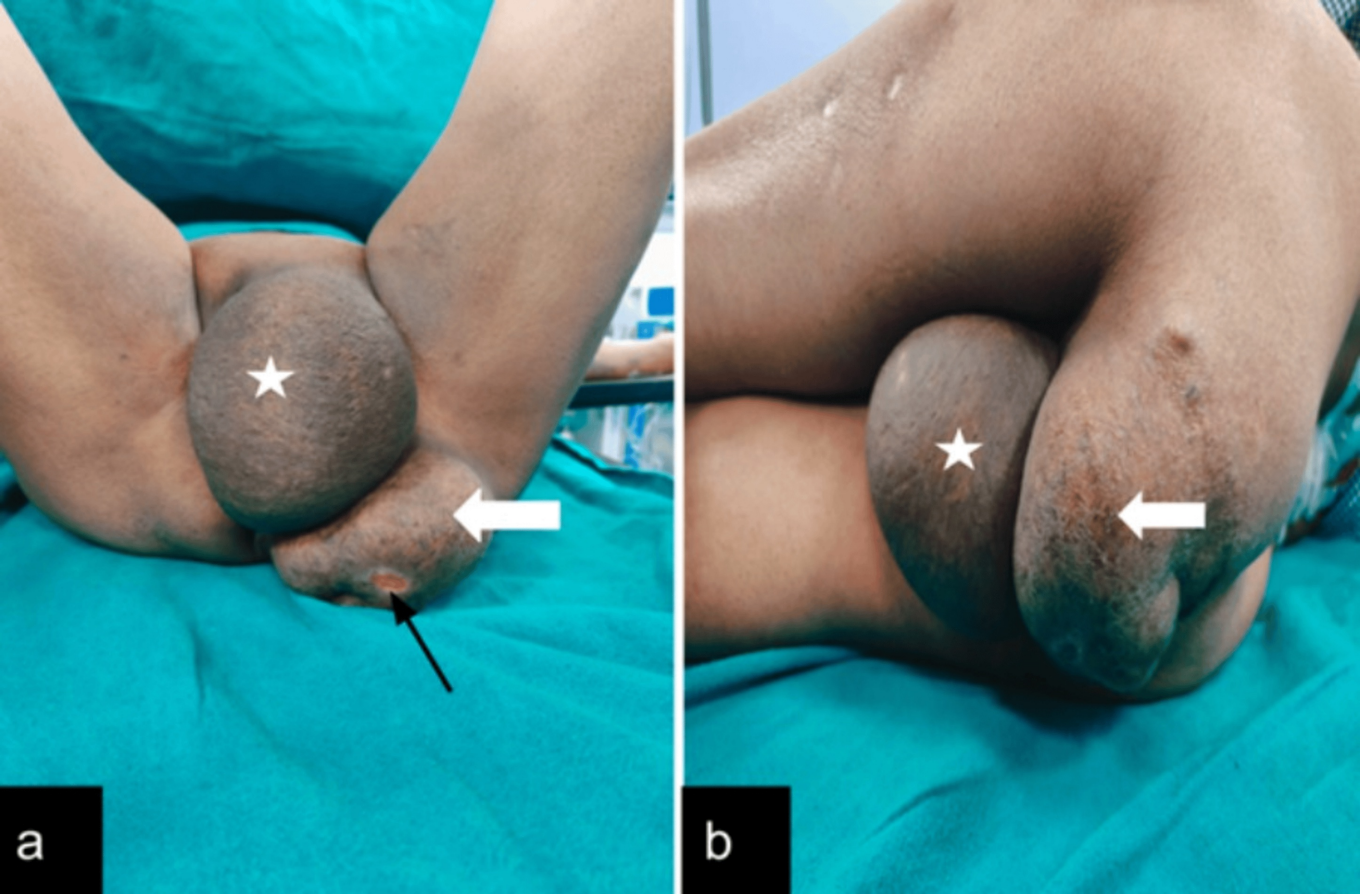
Clinical images (a,b) show globular masses in the perineum lateral to the vagina on the left side (white star symbol) and another mass posterior to it (white arrow) with well-defined margins and smooth surface. Ulceration is seen on the posterior mass (black arrow).

A 47-year-old female presented with a complaint of swelling in the perineal region for 6 years (Figure 1). The patient was apparently asymptomatic six years back, then she noticed swelling in the posterior aspect of the vagina, which was insidious in onset and gradually progressive. The patient had no history of urinary or bowel complaints. No significant weight loss, pain, itching, or redness was noted. Excision of the swelling was done three years ago. Two months later, she noticed swelling in the anterior region of the vagina. Two years back, it was re-excised. These procedures were performed by a local practitioner in a rural setting where histopathology services were not available.

On inspection, there were two swellings; one of size approximately 10cm X 6cm on the left side, lateral to the vagina, and another of size approximately 4cm X 4cm just below the vagina on the left side, posterior to the former swelling. Both masses were globular in shape with well-defined margins and smooth surfaces. A 1 cm X 1 cm ulceration was seen on the posterior swelling. Swelling in the left inguinal region is approximately 2 cm X 2 cm. The masses were of firm consistency, non-reducible, non-tender, with no local rise of temperature. On examination, the mass demonstrated local extension from the vulvovaginal soft tissues and did not show features suggestive of a true herniation. An impulse on coughing was present, which was likely due to transmitted pressure to the lax pelvic soft tissue planes rather than a hernia. No bruit was heard on auscultation. Blood investigations were within normal limits.

**Figure 2:**
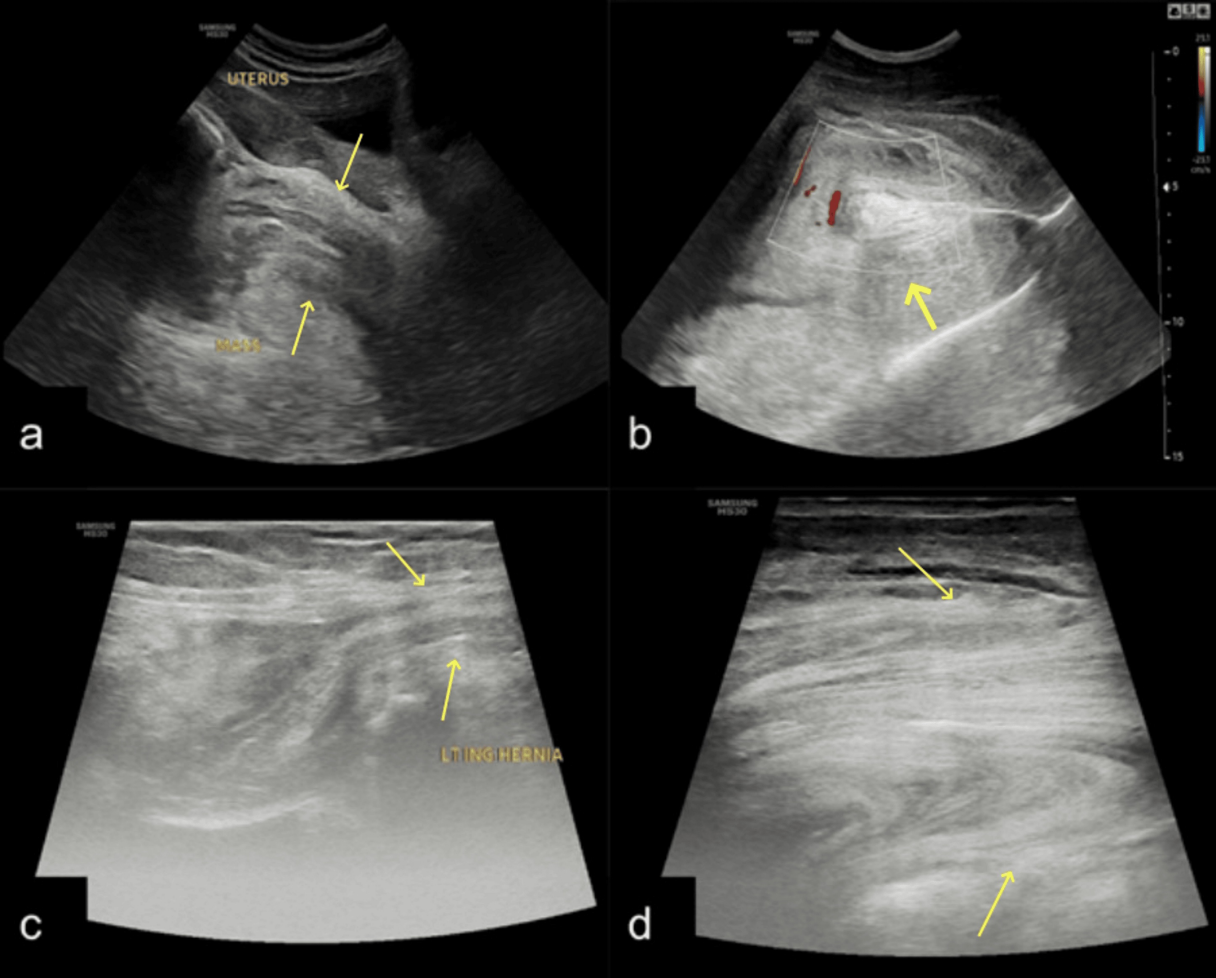
Grayscale ultrasound images obtained using curvilinear (a, b) and linear (c, d) probes show a large, solid, echogenic globular mass in the left inguinolabial region (arrows). The mass demonstrates a characteristic laminated pattern (b, d) and internal vascularity (b). Omentum-like tissue is seen extending into the left inguinal canal (arrows) (c).

**Figure 3:**
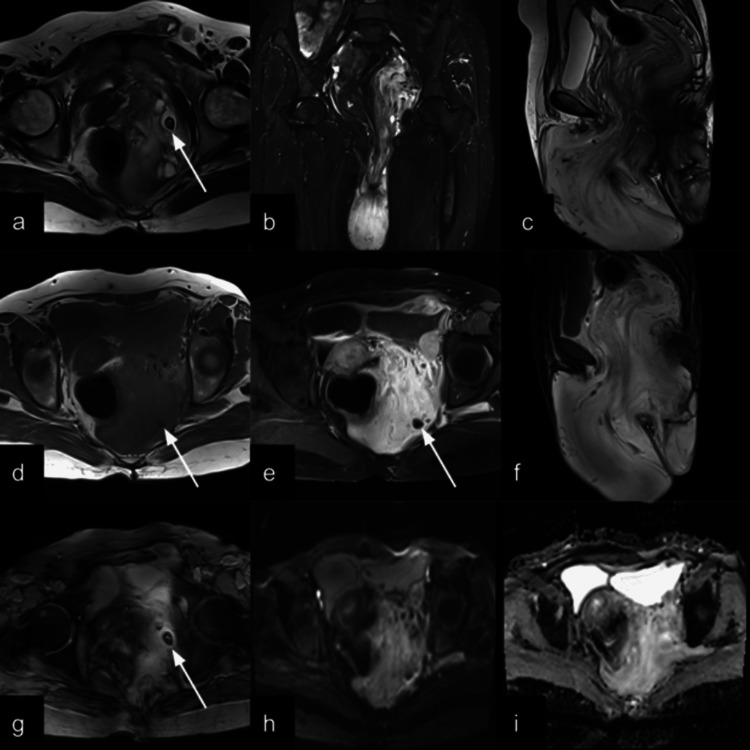
T2-weighted axial (a), STIR coronal (b), and T2-weighted sagittal (c) images show a T2 hyperintense mass with laminated morphology extending from the pelvis into the perineum. Sagittal T2 (c) shows the longitudinal extent of the mass. On the T1-weighted image (d), the mass is isointense. Post-contrast T1 fat-saturated images in axial (e) and sagittal planes (f) showing intense contrast enhancement of the mass. DWI images with B value 800 (h) and the ADC map (i) showing facilitated diffusion (mean ADC value 2.165 × 10⁻³ mm²/s). Axial (a), coronal (b), and sagittal (c) T2-weighted images of the patient show a hyperintense mass with a swirled appearance in the pelvis herniating into the perineum, displacing the hollow pelvic structures. White arrows showing calcification in T2 (a), T1 (d), T1 post-contrast (e), and T2-gradient echo images (g). STIR: short tau inversion recovery; DWI: diffusion-weighted imaging; ADC: apparent diffusion coefficient

**Figure 4:**
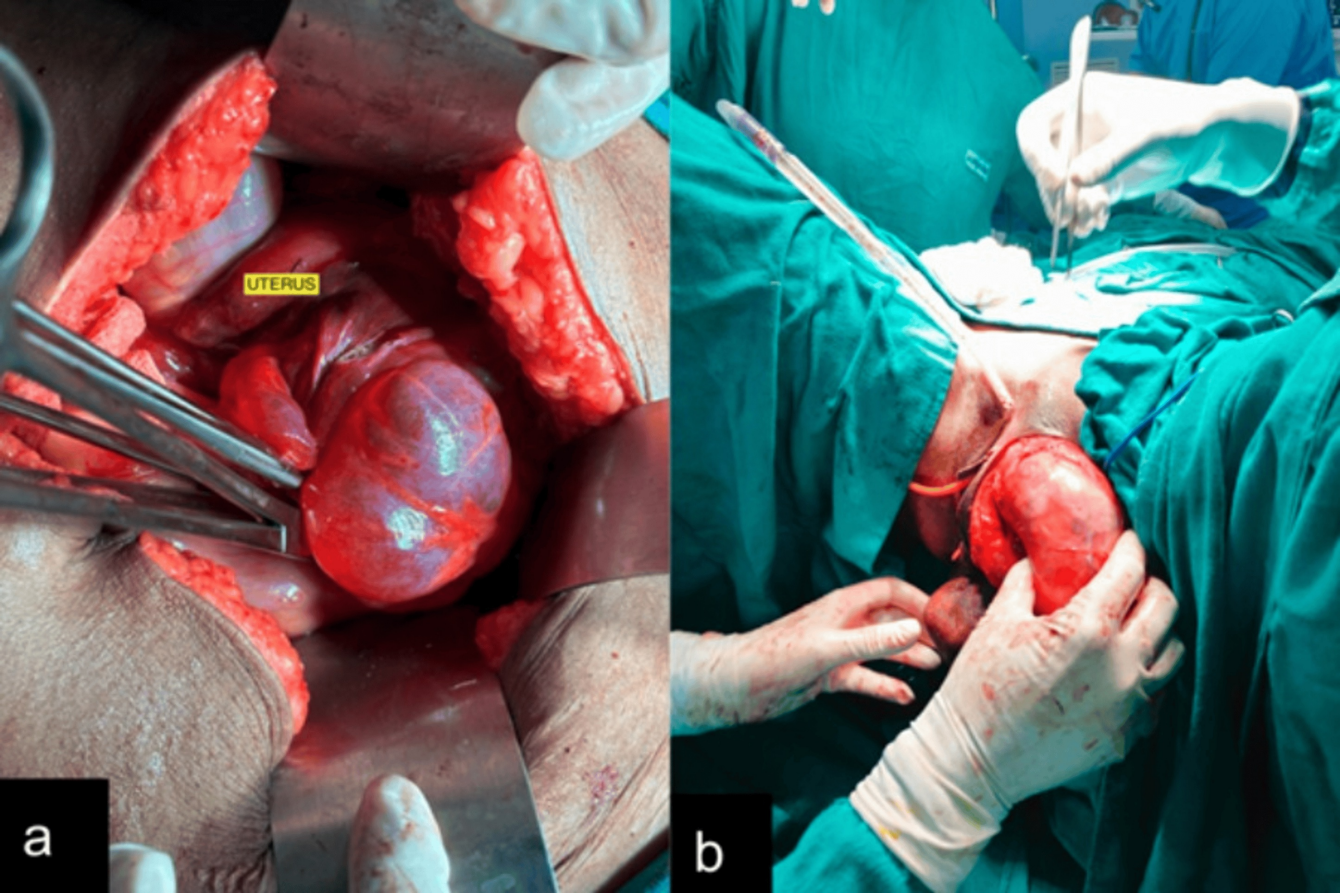
Intraoperative images (a,b) showing the lobulated appearance and perineal herniation of the mass (b)

Based on the history and clinical examination, the differentials of angiomyofibroblastoma, lipoma, and vulvovaginal angiomyxoma were considered. Local site ultrasonography was performed, which showed a large iso-hypoechoic globular mass with a laminated pattern (linear echogenic stripes ) of size ~20 cm X 8 cm in the left inguino-labial region. The lesions showed few linear calcifications and internal vascularity (Figure 2). Similar lesions were also seen in the left gluteal region and posterior to the rectum. MRI was performed to further characterize and discern the extent of the lesion. MRI showed a T2 hyperintense mass with a swirled appearance (Figure 3), herniating into the perineal area, and an imaging diagnosis of vulvovaginal angiomyxoma was made. An exploratory laparotomy with debulking and hysterectomy was performed. Hysterectomy was performed solely because the tumor was inseparable from the uterus, making en bloc excision the safest and most definitive surgical option. Intraoperatively, the tumor was large, bilobed, and present in the left gluteal region, extending from the labia majora to the ischiorectal fossa and further to the posterior aspect of the uterus, lateral pelvic wall, and rectus abdominis region. The tumor was densely adherent to the rectus abdominis, lateral pelvic wall, and uterus (Figure 4).

**Figure 5:**
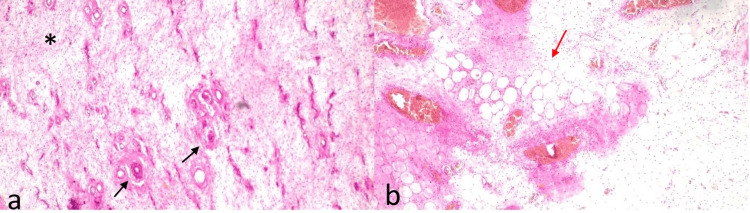
(a, b) Hematoxylin and eosin-stained sections (10× magnification) show a hypocellular benign tumor composed of stellate to spindle cells (*) embedded in abundant myxoid stroma (red arrow in b) with conspicuous, haphazardly arranged dilated capillaries (black arrows in a).

The surgical specimen was sent for histopathology, which showed a hypocelluar benign tumor composed of stellate to spindle cells in a background of abundant myxoid stroma and conspicuous, haphazard dilated capillaries. The tumor was unencapsulated and locally infiltrative (Figure 5). A diagnosis of aggressive angiomyxoma was given.

The postoperative period was uneventful. She was discharged 13 days after surgery and kept on follow-up. The patient is doing well with no local recurrence 12 months post-follow-up.

## Discussion

Aggressive angiomyxoma (AAM) is a rare, benign mesenchymal neoplasm that exhibits a locally infiltrative growth pattern and a high propensity for local recurrence. First described by Steeper and Rosai in 1983, fewer than 350 cases have been reported from 1983 to 2018 [7]. AAM primarily affects women in the reproductive age group, most commonly between the third and fourth decades of life, and arises in the pelvic and perineal regions, including the vulva, vagina, perineum, and pelvis [1].

Despite its histologically benign nature, AAM is characterized by its slow growth, infiltrative margins, and a marked tendency for local recurrence, with recurrence rates reported as high as 30-72%, even years after surgical excision [8]. Distant metastasis is exceedingly rare [9]. The diagnosis is often delayed due to its non-specific clinical presentation, with most patients presenting with a painless, soft, and slowly enlarging mass that may mimic more common pathologies such as Bartholin gland cysts, lipomas, or hernias [9].

On sonographic imaging, AAM typically appears as a hypoechoic soft tissue mass with a laminated, sometimes cystic, appearance [10]. Radiological imaging, particularly magnetic resonance imaging (MRI), plays a crucial role in the preoperative assessment of AAM. MRI typically reveals a well-defined mass with high signal intensity on T2-weighted images due to the myxoid stroma, and iso- to hypointense signal on T1-weighted sequences. A characteristic “swirled” or layered appearance within the lesion on T2-weighted and post-contrast images due to the presence of collagen fibrils in the myxoid tissue in about 83% of patients in an imaging review, which may help in differentiating AAM from other soft tissue tumors [11,12]. Calcifications and cystic changes are found in 6% and 19% of the patients, respectively [12]. The USG and MRI in our case show findings similar to those of previously described cases [10,13,14]. Similar to previous studies, our case shows facilitated diffusion on DWI imaging [12]. Computed tomography (CT) may demonstrate a hypodense, infiltrative lesion, but it is less specific than MRI [15].

Histologically, AAM is composed of hypocellular, myxoid stroma with abundant thin-walled blood vessels and spindle-shaped or stellate cells. Immunohistochemically, the tumor cells are typically positive for vimentin, desmin, smooth muscle actin (SMA), and estrogen and progesterone receptors (ER/PR), indicating hormone sensitivity [4]. This hormone receptor expression has prompted the use of hormonal therapies, such as gonadotropin-releasing hormone (GnRH) agonists, either as adjuvant or primary treatment in unresectable cases [6]. Other pathologies like angiomyofibroblastoma show similar clinicopathological features, but AAM differs by the presence of infiltrative margins and characteristic laminated appearance, which signifies the importance of radiological investigations for more accurate diagnosis [16].

The typical characteristics of an infiltrative mass showing a classic laminated pattern on ultrasound, as seen in our case, which led us to consider aggressive angiomyxoma as the primary diagnosis; however, with a suspicion of an inguinal hernia. Further MRI was conducted to further characterize the lesion's morphology and extent, and typical MRI findings made our diagnosis of AAM consistent. We identified the tissue in the left inguinal region as part of the primary mass. This emphasizes the importance of MRI in evaluating the full extent of aggressive angiomyxoma (AAM), which frequently extends into the perineal and inguinal regions. Ultrasonography alone may lead to misdiagnosis as a herniation. The characteristic imaging and histopathological findings further corroborated our diagnosis of AAM.

Surgical excision with wide margins remains the mainstay of treatment. However, given the lesion’s infiltrative nature and anatomical location, achieving negative margins can be challenging and may lead to functional or cosmetic morbidity. As such, close postoperative follow-up with serial MRI scans is essential to detect early recurrences. The role of radiotherapy and chemotherapy remains limited due to the tumor’s low mitotic activity.

## Conclusions

Aggressive angiomyxoma is a rare, locally infiltrative soft tissue tumor of the vulvovaginal region that poses diagnostic and therapeutic challenges due to its indolent course and high recurrence rate. Awareness of its characteristic imaging features, particularly on MRI, can aid in early diagnosis and surgical planning. Long-term follow-up is essential due to its potential for delayed recurrence. A multidisciplinary approach, including consideration of hormonal therapy in selected cases, may help optimize outcomes.
